# Phytoplasma infection renders cranberries more susceptible to above‐ and belowground insect herbivores

**DOI:** 10.1111/1744-7917.13444

**Published:** 2024-09-15

**Authors:** Cesar Rodriguez‐Saona, Paolo Salazar‐Mendoza, Robert Holdcraft, James Polashock

**Affiliations:** ^1^ P.E. Marucci Center Rutgers University Chatsworth New Jersey United States; ^2^ Department of Entomology and Acarology “Luiz de Queiroz” College of Agriculture University of São Paulo Piracicaba‐SP Brazil; ^3^ Genetic Improvement of Fruits and Vegetables Lab United States Department of Agriculture—Agricultural Research Service Chatsworth New Jersey United States

**Keywords:** consumption, larval plant–insect interactions, oriental beetle, performance, spongy moth, *Vaccinium macrocarpon*

## Abstract

While phytoplasma infections in plants are known to affect their interactions with aboveground herbivores, the impact of different genotypes on these infections and their effects on belowground herbivores remains largely unexplored. In cranberry (*Vaccinium macrocarpon*), infection by the phytoplasma *Candidatus* Phytoplasma sp. subgroup 16SrIII‐Y leads to false blossom disease. This study investigates whether cranberry infection by this phytoplasma affects the performance and feeding behavior of a foliar feeder (spongy moth, *Lymantria dispar*) and a root feeder (oriental beetle, *Anomala orientalis*). Using phytoplasma‐infected and uninfected cranberries of two genotypes (“Ben Lear” and “Crimson Queen”), the survival, growth and consumption of *L. dispar* and *A. orientalis* larvae were measured. To assess the effects on plant morphological and chemical traits, we also examined the impact of phytoplasma infection on shoot and root growth, carbon and nitrogen content, and the levels of defensive compounds such as proanthocyanidins (PACs). Results indicate that larvae of *L. dispar* and *A. orientalis* generally showed larger size and more efficient tissue consumption on infected plants, with these effects varying by cranberry genotype, possibly due to differences in phytoplasma titer. Phytoplasma infection was associated with stunted growth, elevated nitrogen content, and lower PAC levels in both shoots and roots of infected cranberry plants compared to uninfected ones. These findings indicate that phytoplasma infection potentially manipulates plant chemical composition by increasing nutrient levels and decreasing defensive compounds, enhancing herbivore performance both above and belowground. This study sheds light on the intricate interplay among plants, phytoplasma infection, and insect herbivore communities.

## Graphical abstract

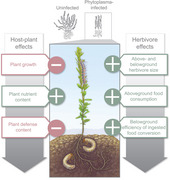

## Introduction

According to the “host manipulation” hypothesis, pathogens have the ability to modify their host's phenotype for their own benefit (Labaude *et al.*, 2015; Heil, 2016; Doherty, 2020). These phenotypic changes can subsequently influence the interactions between plants and insect herbivores (Cory & Hoover, 2006; Mauck *et al.*, 2010; Mauck *et al.*, 2015; Franco *et al.*, 2021). For instance, pathogen‐infected plants may exhibit increased resistance or susceptibility to herbivores due to alterations in plant chemistry (Hammerbacher *et al.*, 2013; Mason *et al.*, 2014; Desurmont *et al.*, 2016; Tamborindeguy *et al.*, 2017; Fernandez‐Conradi *et al.*, 2018).

Among these plant pathogens are phytoplasmas, a type of bacteria lacking a cell wall that infect the plant's phloem and are thus transmitted by phloem‐feeding insects of the Order Hemiptera such as leafhoppers, planthoppers and psyllids (Weintraub & Beanland, 2006; Hogenhout *et al.*, 2008; Bertaccini, 2022). Phytoplasmas can potentially manipulate their hosts, altering both the physical and chemical traits in plants, which can affect their interactions with insect herbivores (Sugio *et al.*, 2011a; Bertaccini, 2022). By secreting protein effectors that interfere with plant cellular processes, phytoplasma infection often suppresses plant defenses, resulting in fitness benefits for both the pathogen and the insect vector (Hogenhout *et al.*, 2008; MacLean *et al.*, 2011; Sugio *et al.*, 2011b; Lu *et al.*, 2014; MacLean *et al.*, 2014; Sugio *et al.*, 2014). For example, Aster Yellows phytoplasma strain Witches’ Broom (16SrI‐B subgroup) lowers plant defenses through a protein effector (SAP11) that benefits the reproduction of its vector, the aster leafhopper *Macrosteles quadrilineatus* Forbes (Hemiptera: Cicadellidae) (Sugio *et al.*, 2011b). Similarly, the reproduction of the Asian citrus psyllid *Diaphorina citri* Kuwayama (Hemiptera: Psyllidae), the vector of *Candidatus* Phytoplasma aurantifolia (16SrII‐B subgroup), which causes Witches’ Broom Disease of Lime, increases when it feeds on infected citrus plants (Queiroz *et al.*, 2016). While it is evident that phytoplasma infection alters plant chemistry, influencing insect vectors, its impact on nonvector organisms, both above‐ and belowground, and whether these effects are consistent across plant genotypes remain largely unexplored.

In cranberry (*Vaccinium macrocarpon* Aiton, Ericaceae), false blossom is a serious threat to the cranberry industry. This disease is caused by *Candidatus* Phytoplasma sp. subgroup 16SrIII‐Y (Lee *et al.*, 2014; Polashock *et al.*, 2017), a phytoplasma that infects all parts of the cranberry plant including leaves, stems, roots and flowers (Trickle *et al.*, 2023) and that is vectored by the blunt‐nosed leafhopper, *Limotettix vaccinii* Van Duzee (Hemiptera: Cicadellidae) (Dobroscky, 1929; Dobroscky, 1931; Lee *et al.*, 2014). The symptoms of this disease include deformed flowers leading to upright flower positioning, sterile ovaries, phyllody, witches’ brooming that causes several branches to appear at the internode, and premature vegetative reddening at the end of the growing season (Dobroscky, 1931), resulting in yield losses (Beckwith & Hutton, 1929).

Recent studies showed that phytoplasma infection of cranberries increases the body size of its insect vector *L. vaccinii* (Pradit *et al.*, 2020; Rodriguez‐Saona *et al.*, 2021) as well as nonvector Lepidopteran herbivores, including spotted fireworm (*Choristoneura parallela* Robinson; Lepidoptera: Tortricidae), Sparganothis fruitworm (*Sparganothis sulfureana* Clemens; Lepidoptera: Tortricidae), and spongy moth (*Lymantria dispar* L.; Lepidoptera: Erebidae) (Pradit *et al.*, 2019a). Notably, larval weights of *L. dispar* were three times bigger when fed on phytoplasma‐infected leaves (Pradit *et al.*, 2019a). This increase in the herbivores’ body size was correlated with higher nitrogen content and reduced phenolic levels, such as proanthocyanidins (PACs), in the aboveground tissues of phytoplasma‐infected cranberry plants compared to uninfected ones (Pradit *et al.*, 2019a). Moreover, phytoplasma infection upregulated the expression of genes associated with nutrient metabolism, while downregulating genes associated with defensive pathways in cranberry leaves (Pradit *et al.*, 2019b). Altogether, these prior studies suggest that *Candidatus* Phytoplasma sp. subgroup 16SrIII‐Y likely manipulates its cranberry host by increasing nutrient availability and suppressing defenses, thus benefiting both the phytoplasma itself and the insect herbivores that feed on it.

In this study, we tested the hypotheses that changes in plant chemistry due to phytoplasma infection affect the performance and feeding behavior of both aboveground and belowground insect herbivores, and that the extent of these changes varies among plant genotypes. Specifically, we aimed to address the following questions: (1) Do the survival, growth and food consumption of an aboveground herbivore (*L. dispar*) and a belowground herbivore (oriental beetle, *Anomala orientalis* Waterhouse; Coleoptera: Scarabaeidae) differ between phytoplasma‐infected and uninfected cranberry plants? (2) Does the effect of phytoplasma infection on these herbivores vary between a native “wild” selection and a recently released cranberry genotype? (3) Does phytoplasma infection alter morphological (size and weight) and chemical (nutrients and defenses) traits in the shoots and roots of cranberry plants? (4) Does the phytoplasma titer differ between tissues (leaves and roots) of the two cranberry genotypes?

## Materials and methods

### Plant material

The collection and propagation of plant material followed methods previously described by Pradit *et al.* (2019a; 2019b; 2020). Dormant phytoplasma‐infected (symptomatic) and uninfected (healthy) cranberry (*V. macrocarpon*) vines were collected from commercial cranberry beds in Chatsworth, New Jersey (USA), during the fall months (November–December) of the year preceding the experiments (i.e., 2018, 2019 and 2022). Two cranberry varieties (referred to herein as “genotypes”) widely grown in the United States were used in the study; these are “Ben Lear,” a native wild genotype selected in 1901 in Berlin, Wisconsin (Eck, 1990), and “Crimson Queen,” a genotype released in 2007 by Rutgers University (New Jersey, USA) (Clark & Finn, 2010). “Crimson Queen” was selected for higher yields, increased anthocyanin content, and enhanced stolon vigor through a cross between the genotypes “Stevens” and “Ben Lear” (Vorsa & Johnson‐Cicalese, 2012). Because “Crimson Queen” was selected for high yield rather than antiherbivore resistance, we expected this genotype to be more susceptible to herbivory than its parental ancestor “Ben Lear.” Also, we expected phytoplasma titers to vary between these genotypes because wild genotypes may be more resistant to phytoplasma infection than cultivated genotypes (Venkataravanappa *et al.*, 2022). The collected plant material was maintained at 10 °C for approximately 3 months.

In February of 2019, 2020, and 2023, both infected and uninfected plants were clonally propagated by transferring 7–7.5 cm stem cuttings to individual 4 cm × 4 cm cells, which were then placed in a greenhouse at 23 ± 2 °C, 60% ± 10% relative humidity (RH), and a 14 h : 10 h (L : D) photoperiod. The plants were grown in a 50 : 50 v/v peat : sand mix, fertilized every 2 weeks starting in March with Jack's water‐soluble 20–20–20 N–P–K (nitrogen–phosphorus–potassium) fertilizer (JR Peters Inc., Allentown, PA, USA) at a rate of 12.3 g per 7 L of water, and were watered daily. Once the cuttings developed roots, groups of five cuttings were transplanted into single (10 cm × 10 cm) pots, with one cutting positioned in each corner and one in the center of the pot. The plants were allowed to grow in the greenhouse for approximately 3 months before being used in experiments. Before starting the experiments, phytoplasma infection was confirmed through visual inspection (e.g., bushy characteristics and short and straight uprights). Additionally, prior to propagation, plant material collected from the field was tested for phytoplasma infection using a nested PCR assay (Lee *et al.*, 2014). Plants to be used as uninfected controls were similarly tested to confirm they were phytoplasma free. At the time of the insect performance and chemical assays (see below), all plants were in the vegetative stage.

### Insects

*Lymantria dispar* (spongy moth) eggs were obtained in late May–June of 2019 from the United States Department of Agriculture Animal and Plant Health Inspection Service (USDA APHIS) Otis Lab (Buzzards Bay, MA, USA) and were maintained at 25 °C until they hatched. Late instar *A. orientalis* (oriental beetle) larvae were collected in May 2020 from soils from infested nursery plants from a commercial nursery in Lumberton, New Jersey (USA), and in May 2023 from turfgrass soils at Rutgers Adelphia Turf Farm in Freehold Township, New Jersey (USA). The larvae were reared to adults individually in 29.6 mL plastic cups containing seeded grass under laboratory conditions at 22 ± 2 °C, 55% ± 5% RH, and a 16 : 8 (L : D) h photoperiod. Upon emergence, males and females were placed together in 473.2 mL deli containers filled halfway with soil from blueberry fields. The containers were regularly checked for newly laid eggs. These eggs were carefully removed from the soil and transferred to polystyrene Petri dishes (35 mm diameter, Falcon brand, Becton‐Dickinson, NJ, USA), lined with a moist piece of filter paper. Each Petri dish contained five eggs that were checked daily for development and considered ready for experiments once they changed to a pearl‐like appearance and became noticeably swollen.

### Experimental design

All experiments were conducted in a greenhouse at the Rutgers P.E. Marucci Center (Chatsworth, NJ, USA), and maintained at a temperature of 23 ± 2 °C, a relative humidity of 60% ± 10%, and a photoperiod of 14 : 10 (L : D) h. The study used a 2 × 2 factorial design, with two levels of “infection” (phytoplasma‐infected and uninfected plants) and two genotypes (“Ben Lear” and “Crimson Queen”). This design resulted in four treatments: “uninfected Ben Lear,” “infected Ben Lear,” “uninfected Crimson Queen,” and “infected Crimson Queen.” We used the “pot” as the experimental unit for these experiments because they were our independent data points.

Following the four treatments, we used the potted plants to assess the effects of phytoplasma infection and plant genotype on the performance (survival and weight) and consumption rates of *L. dispar* and *A. orientalis* larvae (see below). Additionally, we measured shoot and root growth, carbon/nitrogen (C/N) ratios as a proxy for nutritional content, PAC levels as a proxy for defense content, and phytoplasma titers in both shoots and roots (see below).

### Effects of phytoplasma infection and genotype on L. dispar

In June–July 2019, experiments were conducted to assess *L. dispar* larval performance and consumption on phytoplasma‐infected and uninfected cranberry leaves. Ten pots were randomly assigned to each of the four treatments. In each pot, one random plant out of the five was covered with an 18 cm × 42 cm × 48 cm gauze bag (Temkin International; Springville, UT, USA) and received three 1st instars of *L. dispar*. The experiment was repeated twice (*n* = 20 replicates per treatment).

After 7 d, larval mortality and weight were assessed. The weight of each larva was measured using a high precision balance (Sartorius BP211D; Göttingen, Germany). The number of damaged leaves was estimated by counting the leaves with visible signs of larval feeding. The damaged leaves were then scanned and the images analyzed using ImageJ Software version 1.52a (Rasband, 2020) to calculate consumed leaf area (cm^2^).

### Effects of phytoplasma infection and genotype on A. orientalis

In July–August 2020 and 2023, experiments were conducted to assess *A. orientalis* larval performance and consumption on phytoplasma‐infected and uninfected cranberry roots. Fifteen pots (in 2020) and 24 pots (in 2023) were randomly assigned to each of the four treatments (*n* = 15 and 24 replicates per treatment). Three *A. orientalis* eggs were placed in each pot, positioned next to individual cuttings at a depth of approximately 4 cm. After 1.5 months, the soil in the pots was carefully examined for larvae, and larval mortality and weights were assessed. Individual larvae were weighed using a Mettler‐Toledo AC245 balance (Mettler‐Toledo, Columbus, OH, USA).

In 2020, *A. orientalis* root consumption was calculated by carefully washing and weighing the roots of plants from each pot using a Mettler AE 50 balance. The roots from an additional 10 pots per treatment but without grubs were also weighed. Root consumption by *A. orientalis* larvae was estimated by subtracting the average root weight of plants in pots with larvae from those without larvae.

### Effects of phytoplasma infection and genotype on shoot and root growth

In July 2020, 10 pots were randomly selected from each of the four treatments for shoot and root growth measurements (*n* = 10 replicates per treatment). The length of all vines within each pot was measured, and the mean lengths were calculated to determine the size of each plant. Afterward, the shoots and roots of all plants in each pot were removed and weighed using an Ohaus Scout Pro balance (Ohaus Corp., Pine Brook, NJ, USA). After weighing, the plant material was placed in paper bags and allowed to air dry for approximately 2 months. The dry weights of shoots and roots were determined using the Mettler AE 50 balance (Mettler‐Toledo, Columbus, OH, USA) and a mean was calculated for each pot.

### Effects of phytoplasma infection and genotype on nitrogen and carbon/nitrogen (C/N) ratios

Given that nitrogen content in plants is a limiting factor for insect herbivores (Mattson, 1980), C/N ratios can serve as valuable indicators of food quality (Royer *et al.*, 2013). In July 2020, two random plants from each of 10 randomly selected pots for each of the four treatments were chosen for nitrogen and carbon analyses (*n* = 10 replicates per treatment). The shoots and roots of the selected plants were placed in paper bags and allowed to dry. The dried samples (1.5 g each) were then sent to the Penn State University Agricultural Analytical Service Laboratory (http://agsci.psu.edu/aasl). Total nitrogen and carbon levels were determined by combustion using an Elementar Vario Max N/C Analyzer (Pella, 1990; Horneck & Miller, 1998) and used to calculate C/N ratios.

### Effects of phytoplasma infection and genotype on PAC levels

We measured PAC levels in both cranberry leaves and roots as a proxy of antiherbivore defenses. PACs, also known as condensed tannins, are widespread polymeric flavan‐3‐ols that bind to proteins and exhibit antiherbivore and antimicrobial properties (Dixon & Sarnala, 2020). In July 2023, one random plant from each of eight randomly selected pots for each of the four treatments was selected for PAC analysis (*n* = 8 replicates per treatment). PAC analysis followed the methods outlined by de Lange *et al.* (2019). For each treatment, shoots and roots were harvested and stored at −20 °C before extraction. Frozen tissues (approximately 400  mg) were ground with a mortar and pestle and transferred to microcentrifuge tubes. A solvent consisting of 40 : 40 : 20 : 0.1 acetone : methanol : water : ascorbic acid (Sigma‐Aldrich, St. Louis, MO, USA) was added (1 mL), and the samples were sonicated at room temperature for 10 min, vortexed for 5 min, and then centrifuged for 8 min at 6700 × *g*. The resulting supernatant was analyzed using a 4‐dimethylaminocinnamaldehyde (DMAC) spectrophotometric assay. To prepare the methanol‐based DMAC reagent, 0.1 g of DMAC (Sigma‐Aldrich, St. Louis, MO, USA) was weighed and added to 25 mL of HCl (37%) (Fisher Scientific, Hampton, NH, USA), and then brought up to a volume of 100 mL with methanol. Samples were diluted with 100% methanol to obtain absorbance readings in the appropriate range. Mixtures of 0.25 mL of the sample solution with 1.75 mL of DMAC reagent were prepared in a cuvette with a path length of 1 cm. The maximum absorbance (640 nm) of the PAC‐DMAC conjugate was recorded after 9 min of reaction. Absorbance was measured using a ultraviolet‐visible spectrophotometer (Genesys 10S UV‐VIS, Thermo Scientific, Wilmington, DE, USA). Total PAC concentration was calculated based on a standard curve prepared by reacting DMAC with PACs extracted from cranberry fruits.

### Effects of plant tissue and genotype on phytoplasma titer

Since differences in the phytoplasma titer in infected plants may affect insect performance and feeding behavior, we measured the relative phytoplasma quantity in tissues (leaves and roots) of infected plants of both cranberry genotypes (“Ben Lear” and “Crimson Queen”) using qPCR. About 50 mg of leaf tissues and 50 mg of washed and dried root tissues from infected and uninfected plants were collected and stored in a −70 °C freezer until processed. DNA was isolated from the leaf tissue using a modified CTAB extraction procedure (Daverdin *et al.*, 2017). The root tissues were lyophilized overnight in a benchtop freeze dryer (FreeZone 2.5 Liter, Labconco, Kansas City, MO, USA). DNA was extracted from the lyophilized root tissues using the Monarch Genomic DNA Purification kit (New England Biolabs, Ipswich, MA, USA). Extracted DNA was quantified using the Qubit BR Assay Kit (ThermoFisher Scientific, Waltham, MA, USA). DNA samples extracted from leaves were diluted to 50 ng/*µ*L and those from roots were diluted to 10 ng/*µ*L.

The target for relative quantity determination of the phytoplasma was the *secY* gene (Bagadia *et al.*, 2013). Plant reference genes used as endogenous controls were *actin* and *RNA Helicase 8* (Rodriguez‐Saona *et al.*, 2013). All primers and probes were designed using sequence data generated in our lab (J. Polashock, unpublished) and the PrimerQuest Tool (Integrated DNA Technologies, Coralville, IA, USA). All probes were labeled with 6‐FAM and contained the Black Hole Quencher 1 (Integrated DNA Technologies, Coralville, IA, USA). The primers and probes were purchased as a PrimeTime Standard qPCR assay (Integrated DNA Technologies, Coralville, IA, USA) and are shown in Table S1. Assays were diluted as per manufacturer's guidelines to yield a final 1× concentration of 500 nmoles of each (F and R) primer and 250 nmoles of probe. Reactions utilized PrimeTime Gene Expression Master mix (Integrated DNA Technologies, Coralville, IA, USA). Each 20 *µ*L reaction contained: 10 *µ*L of 2× PrimeTime mastermix, 1 *µ*L of diluted primer/probe mix, 50 ng of DNA and nuclease free water to 20 *µ*L. All reactions were run on an Applied Biosystems QuantStudio 5 Real‐Time PCR System (ThermoFisher) using the following parameters: Initial incubation of 50 °C for 2 min followed by 95 °C for 10 min and then 40 cycles of 95 °C for 15 s and 60 °C for 1 min. There were five biological replicates for each sample, and three technical replicates were run for each biological replicate. The relative quantification (RQ) of the target (*secY*) gene was calculated using the endogenous control genes (*actin* and *RNA Helicase 8*) for normalization and the 2^−∆∆Ct^ method with the DataAssist Software v3.01 (ThermoFisher).

### Statistical analysis

Prior to statistical analyses, the data were averaged for each pot, which served as the experimental unit. All data were checked for normality using the Shapiro–Wilk test, and homogeneity of variances using Levene's test before statistical analyses. The efficiency of ingested food conversion (ECI) was computed using the formula ECI = (weight of larvae/weight of food ingested × 100%) for both *L. dispar* and *A. orientalis* larvae. Prior to conducting univariate analysis, we performed a 2 × 2 Multivariate Analysis of Variance (MANOVA) to investigate the impacts of infection level (infected versus uninfected) and plant genotype (“Ben Lear” versus “Crimson Queen”), as well as their interaction, on larval performance (weight and survival) for both *L. dispar* and *A. orientalis* because these parameters are not independent. Because the study on *A. orientalis* was conducted over 2 years, we included “year” as a block effect in the model.

Next, larval weights, larval consumption, and ECI, as well as shoot and root size (length), dry weights, water content, nitrogen content, C/N ratio, and total PACs were subjected to Generalized Linear Models (GLM) with a gamma distribution (log link), that included a 2 × 2 factorial interaction for the level of infection and genotype. Percent larval survival and the number of damaged leaves were analyzed using a GLM with a quasibinomial (log link) and quasipoisson (log link) distribution, respectively. To compare the phytoplasma titer (RQ values) between plant tissues (leaves and roots) and genotypes, we also performed a GLM with a gamma distribution (log link). *Post hoc* tests were conducted using Tukey's method (*P* < 0.05) for each factor.

All data were analyzed using RStudio software version 2023.09.0 (R Core Team, 2022), with the “*hnp*,” “*emmeans*,” and “*multcomp*” packages, except for MANOVAs that were performed using Minitab version 17 (Minitab Inc., State College, PA, USA).

## Results

### Effects of phytoplasma infection and genotype on L. dispar

According to MANOVA, phytoplasma infection (Wilks’ Lambda = 0.55; *F* = 30.636; df = 3, 75; *P* < 0.001), genotype (Wilks’ Lambda = 0.806; *F* = 9.027; df = 3, 75; *P* < 0.001), and the interaction between infection and genotype (Wilks’ Lambda = 0.881; *F* = 5.061; df = 3, 75; *P* = 0.009) had a significant effect on *L. dispar* larval performance.

**Fig. 1:**
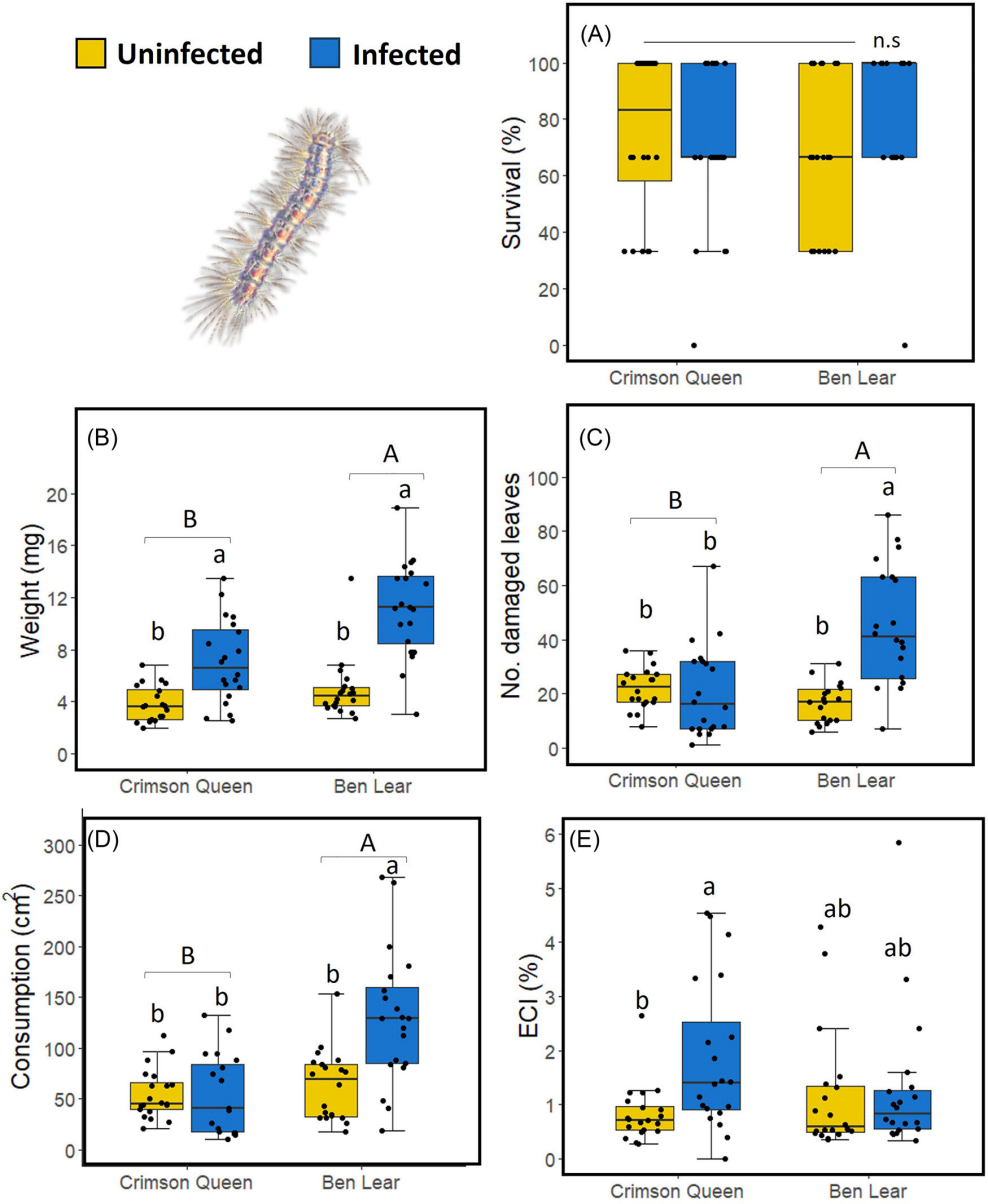
Effects of phytoplasma infection on *Lymantria dispar* (spongy moth) larval performance and food utilization in two cranberry genotypes (“Crimson Queen” and “Ben Lear”). Box plots of: (A) percent larval survival; (B) larval weights; (C) number of damaged leaves; (D) leaf consumption; (E) efficiency of ingested food conversion (ECI). The box plots indicate the first quartile (bottom of the box), third quartile (top of the box), median (line in the box), smallest nonoutlier (lower whisker), largest nonoutlier (upper whisker), and outliers (black circles outside the boxes). ECI was calculated as: weight of larvae/weight of food ingested ×100%. Different lowercase letters indicate significant differences between phytoplasma‐infected and uninfected plants across genotypes (*P* ≤ 0.05); different uppercase letters indicate significant differences between genotypes (*P* ≤ 0.05); n.s = no significant differences (*P* > 0.05). *n* = 20.

Neither phytoplasma infection, genotype, nor the interaction between infection and genotype affected *L. dispar* larval survival (Table 1; Fig. 1A). However, the larvae of *L. dispar* were two times bigger (Fig. 1B), damaged 70% more leaves (Fig. 1C), consumed 56% more leaf material (Fig. 1D), and had 51% higher ECI (Fig. 1E) on phytoplasma‐infected than uninfected plants (Table 2). The larvae were also bigger (Fig. 1B), damaged more leaves (Fig. 1C), and consumed more leaf material (Fig. 1D) on “Ben Lear” than on “Crimson Queen” (Table 2). There was, however, a significant infection‐by‐genotype effect on the number of damaged leaves and leaf consumption (Table 2), indicating that the effect of phytoplasma infection on *L. dispar* feeding behavior was influenced by genotype (Fig. 1C, D).

**Table 1:** Generalized linear model output for the effects of phytoplasma infection, genotype and the interaction between infection and genotype on the performance and food utilization parameters of the foliar feeder *Lymantria dispar* (spongy moth) and the root feeder *Anomala orientalis* (oriental beetle) in cranberries

			Infection^‡^			Genotype^‡^			Infection × Genotype^‡^		
Source of variation			*χ^2^*	df	*P*	*χ^2^*	df	*P*	*χ^2^*	df	*P*
*Lymantria dispar*	Survival		0.865	1	0.352	0.442	1	0.506	3.231	1	0.072
	Weight		61.094	1	**< 0.001**	14.446	1	**< 0.001**	1.373	1	0.241
	No. damaged leaves		17.939	1	**< 0.001**	9.249	1	**0.002**	16.761	1	**< 0.001**
	Consumption		8.079	1	**0.004**	14.921	1	**< 0.001**	7.976	1	**0.040**
	ECI^†^		5.997	1	**0.014**	0.067	1	0.79	3.767	1	0.052
*Anomala orientalis*	Survival	2020	14.416	1	**< 0.001**	0.027	1	0.867	12.661	1	**< 0.001**
		2023	51.753	1	**< 0.001**	5.848	1	0.051	0.003	1	0.953
	Weight	2020	34.142	1	**< 0.001**	6.555	1	**0.010**	0.443	1	0.505
		2023	28.947	1	**< 0.001**	0.010	1	0.919	0.521	1	0.470
	Consumption		413.08	1	**< 0.001**	47.07	1	**< 0.001**	0.110	1	0.736
	ECI^†^		71.62	1	**< 0.001**	6.659	1	**< 0.001**	0.111	1	0.738

^†^ Efficiency of ingested food conversion (ECI) = weight of larvae/weight of food ingested × 100%.

^‡^ Significant effects are highlighted in bold (*P* ≤ 0.05).

**Table 2:** Generalized linear model output for the effects of phytoplasma infection, genotype and the interaction between infection and genotype on shoot and root parameters in cranberries

		Infection^‡^			Genotype^‡^			Infection × Genotype^‡^		
Source of variation^†^		*χ^2^*	df	*P*	*χ^2^*	df	*P*	*χ^2^*	df	*P*
Shoots (leaves)	Length	35.655	1	**< 0.001**	51.833	1	**< 0.001**	0.128	1	0.720
	Dry weight	22.026	1	**< 0.001**	23.227	1	**< 0.001**	8.601	1	**0.003**
	Nitrogen	81.231	1	**< 0.001**	83.032	1	**< 0.001**	15.605	1	**< 0.001**
	C/N ratio	87.789	1	**< 0.001**	84.409	1	**< 0.001**	13.539	1	**< 0.001**
	Total PACs	20.031	1	**< 0.001**	0.019	1	0.888	0.365	1	0.545
Roots	Dry weight	8.04	1	**0.005**	1.312	1	0.252	10.606	1	**0.001**
	Nitrogen	4.185	1	**0.040**	1.463	1	0.226	1.343	1	0.246
	C/N ratio	0.224	1	0.635	1.113	1	0.291	0.199	1	0.655
	Total PACs	15.705	1	**< 0.001**	0.282	1	0.594	1.125	1	0.288

^†^ C/N = carbon/nitrogen; PACs = proanthocyanidins.

^‡^ Significant effects are highlighted in bold (*P* ≤ 0.05).

### Effects of phytoplasma infection and genotype on A. orientalis

According to MANOVA, phytoplasma infection (Wilks’ Lambda = 0.526; *F* = 63.925; df = 2, 142; *P* < 0.001) had a significant effect on *A. orientalis* larval performance. However, there was no effect of genotype on *A. orientalis* larval performance (Wilks’ Lambda = 0.971; *F* = 2.134; df = 2, 142; *P* = 0.122), and a marginal interaction effect of infection and genotype (Wilks’ Lambda = 0.959; *F* = 2.967; df = 2, 142; *P* = 0.055). Because there was a significant effect of “year” (Wilks’ Lambda = 0.386; *F* = 112.684; df = 2, 142; *P* < 0.001), *A. orientalis* performance parameters were analyzed separately for each year.

**Fig. 2:**
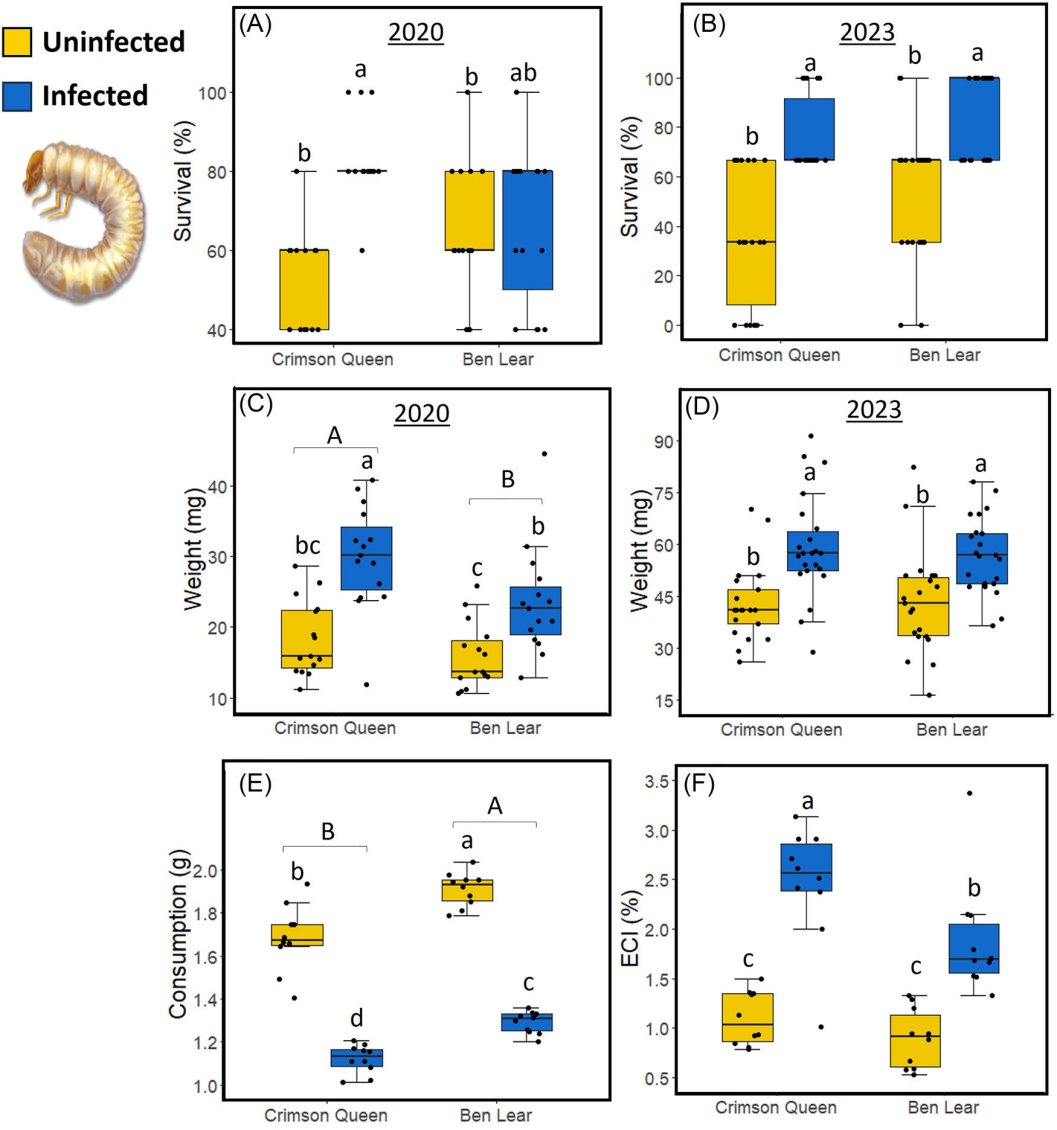
Effects of phytoplasma infection on *Anomala orientalis* (oriental beetle) larval performance and food utilization in two cranberry genotypes (“Crimson Queen” and “Ben Lear”). Box plots of: (A) percent larval survival in 2020; (B) percent larval survival in 2023; (C) larval weights in 2020; (D) larval weights in 2023; (E) root consumption; (F) efficiency of ingested food conversion (ECI). The box plots indicate the first quartile (bottom of the box), third quartile (top of the box), median (line in the box), smallest nonoutlier (lower whisker), largest nonoutlier (upper whisker), and outliers (black circles outside the boxes). ECI was calculated as: weight of larvae/weight of food ingested × 100%. Different lowercase letters indicate significant differences between phytoplasma‐infected and uninfected plants across genotypes (*P* ≤ 0.05); different uppercase letters indicate significant differences between genotypes (*P* ≤ 0.05). *n* = 15 in 2020 and *n* = 24 in 2023.

Survival of *A. orientalis* larvae was 15%–30% higher on phytoplasma‐infected than uninfected plants (Table 1; Fig. 2A, B). However, this effect was influenced by genotype in 2020 (Table 1; Fig. 2A). The larvae of *A. orientalis* exhibited a 37%–55% increase in weight (Table 1; Fig. 2C, D), while consuming 50% less root tissues on phytoplasma‐infected plants compared to uninfected ones (Table 1; Fig. 2E). Consequently, this led to 2.2 times higher ECI on phytoplasma‐infected plants (Table 1; Fig. 2F). The larvae of *A. orientalis* were also bigger in 2020 (Table 1; Fig. 2C) and had higher ECI (Table 1; Fig. 2F), while consuming less root material (Table 1; Fig. 2E), on “Crimson Queen” than “Ben Lear.” No interaction effect was observed between infection and genotype on larval weights and root consumption (Table 1), indicating that genotype has limited influence on the effects of phytoplasma infection on *A. orientalis* performance and feeding behavior.

### Effects of phytoplasma infection and genotype on shoot and root growth

**Fig. 3:**
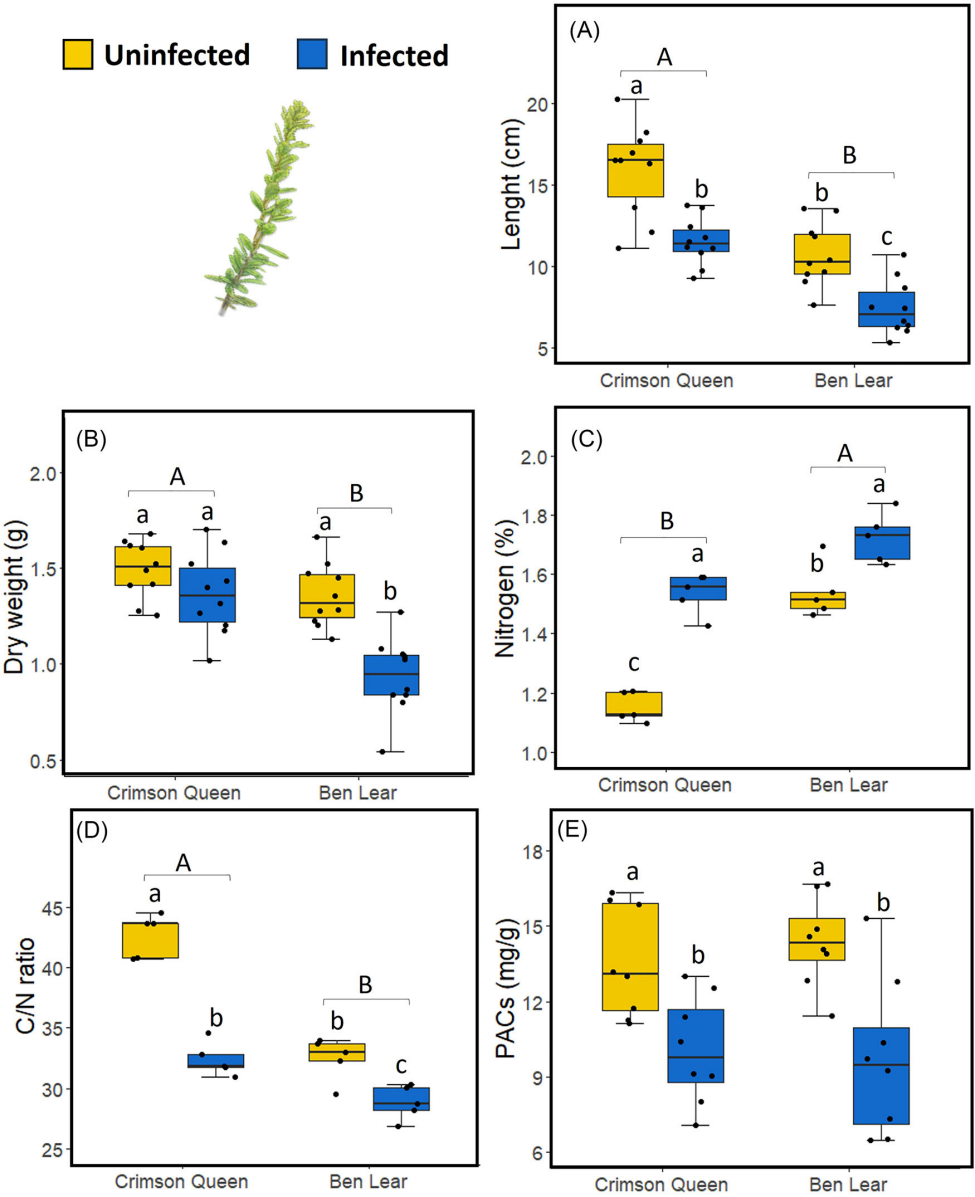
Effects of phytoplasma infection on shoot parameters in two cranberry genotypes (“Crimson Queen” and “Ben Lear”). Box plots of: (A) length of vines; (B) dry weights of vines; (C) nitrogen content in leaves; (D) carbon/nitrogen (C/N) ratios in leaves; (E) levels of proanthocyanidins (PACs) in leaves. The box plots indicate the first quartile (bottom of the box), third quartile (top of the box), median (line in the box), smallest nonoutlier (lower whisker), largest nonoutlier (upper whisker), and outliers (black circles outside the boxes). Different lowercase letters indicate significant differences between phytoplasma‐infected and uninfected plants across genotypes (*P* ≤ 0.05); different uppercase letters indicate significant differences between genotypes (*P* ≤ 0.05). *n* = 10, except for PACs where *n* = 8.

Shoot length and weight were significantly affected by phytoplasma infection and genotype (Table 2). Uninfected plants were 40% longer (Fig. 3A) and 25% heavier (Fig. 3B) than phytoplasma‐infected plants. Also, “Crimson Queen” was longer and heavier than “Ben Lear” (Fig. 3A, B). There was, however, a significant interaction effect between infection and genotype on shoot weight (Table 2), such that only the shoot weight of “Ben Lear” was affected by infection (Fig. 3B).

**Fig. 4:**
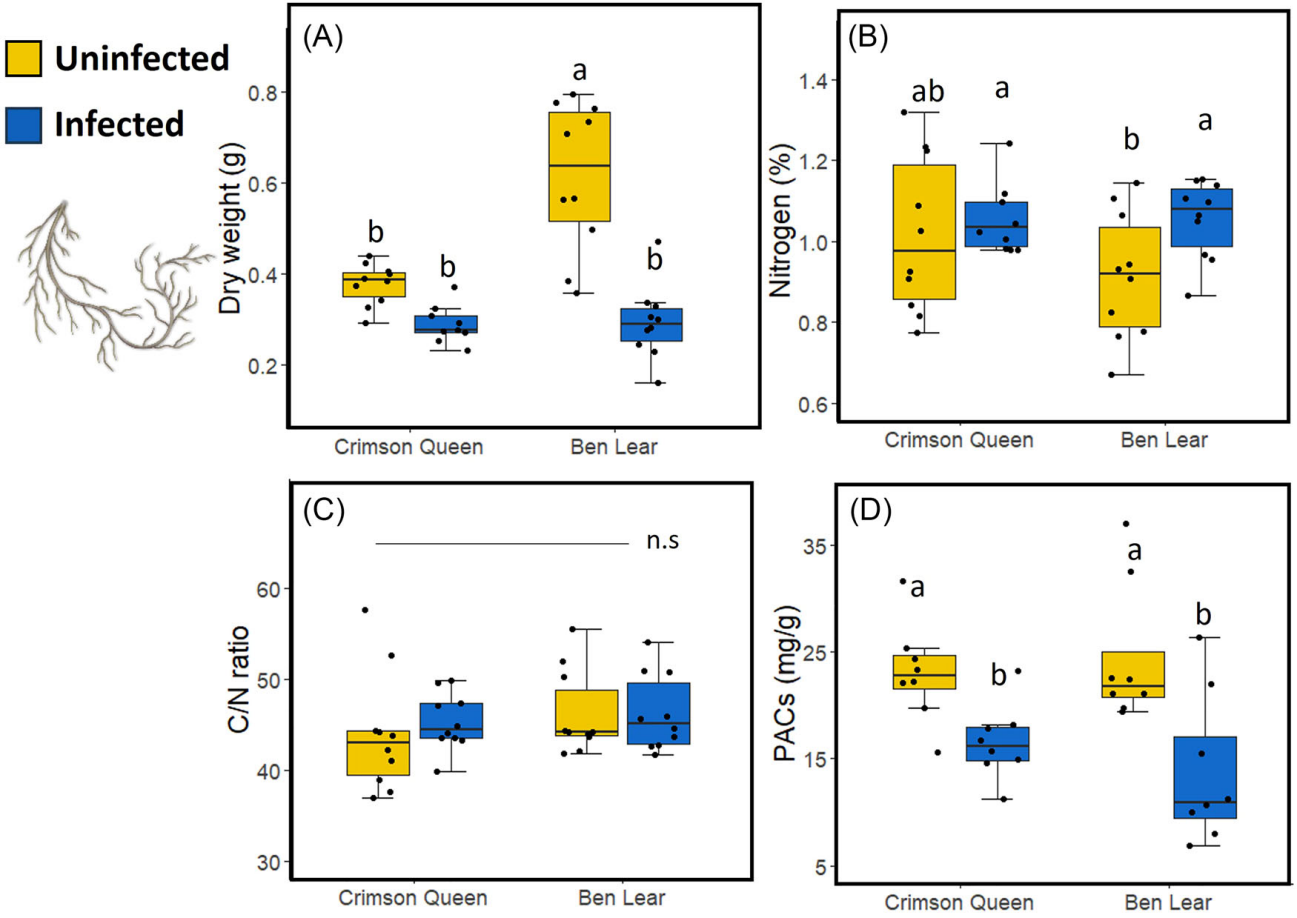
Effects of phytoplasma infection on root parameters in two cranberry genotypes (“Crimson Queen” and “Ben Lear”). Box plots of: (A) dry weights of roots; (B) nitrogen content in roots; (C) carbon/nitrogen (C/N) ratios in roots; (D) levels of proanthocyanidins (PACs) in roots. The box plots indicate the first quartile (bottom of the box), third quartile (top of the box), median (line in the box), smallest nonoutlier (lower whisker), largest nonoutlier (upper whisker), and outliers (black circles outside the boxes). Different lowercase letters indicate significant differences between phytoplasma‐infected and uninfected plants across genotypes (*P* ≤ 0.05); n.s = no significant differences (*P* > 0.05). *n* = 10, except for PACs where *n* = 8.

Root weight was also affected by phytoplasma infection but not by genotype (Table 2), with roots being 43% heavier in uninfected than infected plants (Fig. 4A). However, there was a significant interaction effect between infection and genotype on root weight (Table 2), with infection reducing the root weights of “Ben Lear” but not those of “Crimson Queen” (Fig. 4A).

### Effects of phytoplasma infection and genotype on nitrogen and carbon/nitrogen (C/N) ratios

Nitrogen content and C/N ratios in leaves were significantly affected by phytoplasma infection and genotype (Table 2). Phytoplasma‐infected leaves had higher percent nitrogen (Fig. 3C) and lower C/N ratios (Fig. 3D) than uninfected plants. Also, leaves of “Ben Lear” had higher percent nitrogen and lower C/N ratios than “Crimson Queen” (Fig. 3C, D). There was, however, a significant interaction effect between infection and genotype on leaf nitrogen content and C/N ratios (Table 2), with the effect of infection on nitrogen content and C/N ratios being stronger in “Crimson Queen” than in “Ben Lear” (Fig. 3C, D).

Nitrogen content, but not C/N ratios, in roots was also affected by phytoplasma infection (Table 2; Fig. 4B, C). Nitrogen levels were higher in roots of phytoplasma‐infected than uninfected plants (Fig. 4B). There was no effect of genotype or an interaction effect between infection and genotype on nitrogen content and C/N ratios in roots (Table 2).

### Effects of phytoplasma infection and genotype on proanthocyanidin levels

Total PACs were 50% and 67% lower in leaves (Fig. 3E) and roots (Fig. 4D), respectively, of phytoplasma‐infected than uninfected plants (Table 2). There was no effect of genotype or an interaction effect between infection and genotype on total PAC levels in both leaves and roots (Table 2).

### Effects of plant tissue and genotype on phytoplasma titer

**Fig. 5:**
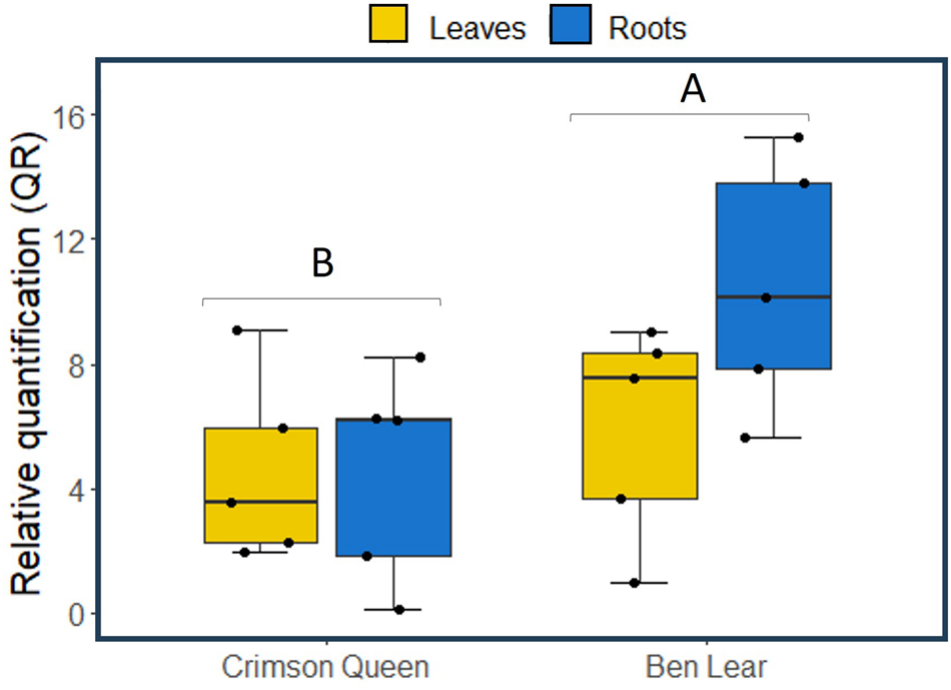
Relative quantification (RQ) of the target (*secY*) gene in leaves and roots of the cranberry genotypes “Ben Lear” and “Crimson Queen.” The box plots indicate the first quartile (bottom of the box), third quartile (top of the box), median (line in the box), smallest nonoutlier (lower whisker), and largest nonoutlier (upper whisker). *Actin* and *RNA Helicase 8* were used as endogenous control genes. The data were calculated based on five biological replicates.

There were differences in phytoplasma titer between cranberry genotypes (*χ* ^2^ = 4.025; df = 1; *P* = 0.045), with infected “Ben Lear” plants having a higher titer than infected “Crimson Queen” plants (Fig. 5). There were, however, no differences between leaves and roots (*χ* ^2^ = 1.097; df = 1; *P* = 0.295), nor was there an interaction effect between plant tissue and genotype (*χ* ^2^ = 1.157; df = 1; *P* = 0.282), in phytoplasma titer. The *secY* target gene did not amplify in any of the controls (uninfected plants) at the conclusion of the experiments.

## Discussion

There is limited knowledge about the impact of phytoplasma infection on insect communities, especially regarding belowground herbivores. Additionally, it remains uncertain whether these effects vary based on the host‐plant genotype. This study demonstrates that phytoplasma infection increases the susceptibility of cranberry to both above‐ and belowground herbivores. We also found that phytoplasma infection increases nitrogen levels and reduces secondary metabolites, such as proanthocyanidins, in cranberry shoots and roots, which may explain the increased performance and feeding by leaf and root feeders on infected plants. Although phytoplasma infection increased susceptibility to *L. dispar* and *A. orientalis* in both genotypes “Ben Lear” and “Crimson Queen,” there were instances where host‐plant genotype influenced the magnitude of the effects of phytoplasma infection on the herbivore and the plant traits.

### Do phytoplasma infection and genotype affect an aboveground herbivore?

In a previous study, we demonstrated that *L. dispar* larvae grow larger and cause more damage to leaves on phytoplasma‐infected cranberry plants compared to uninfected plants (Pradit *et al.*, 2019a). These earlier experiments were conducted using a single genotype, “Crimson Queen.” In this study, we expanded our investigation to include both the “Crimson Queen” and “Ben Lear” genotypes infected by phytoplasma (Fig. 1B). Our findings confirmed that *L. dispar* larvae exhibit increased growth in both genotypes when infected by phytoplasma. Notably, the impact of phytoplasma infection on larval growth was more pronounced in the “Ben Lear” genotype compared to “Crimson Queen,” possibly attributable to the higher phytoplasma titer in “Ben Lear” (Fig. 5). Furthermore, our observations revealed a significant effect of phytoplasma infection on leaf comsumption by *L. dispar* larvae in “Ben Lear,” whereas no such effect was observed in “Crimson Queen” (Fig. 1C, D). These findings led to a higher efficacy of ECI in phytoplasma‐infected “Crimson Queen” than in “Ben Lear” (Fig. 1E).

Together, our previous and current studies show that phytoplasma infection increases the susceptibility of cranberries to the aboveground herbivore *L. dispar*. However, the magnitude of the phytoplasma titer and the effects of infection on larval performance and consumption were determined by the genotype. Since breeding for high‐yielding genotypes can reduce defensive traits (Rodriguez‐Saona *et al.*, 2011; Meyer *et al.*, 2012; Chen *et al.*, 2015; Moreira *et al.*, 2018), which can make them more susceptible to herbivory compared to their wild counterparts (Whitehead *et al.*, 2017; Gaillard *et al.*, 2018; Salazar‐Mendoza *et al.*, 2024), we expected “Ben Lear” to have higher antiherbivore resistance than “Crimson Queen.” Contrary to this expectation, uninfected “Ben Lear” and “Crimson Queen” exhibited equal resistance to *L. dispar*. However, the phytoplasma titer was higher and infection more strongly reduced resistance in “Ben Lear” than in “Crimson Queen.” For a more comprehensive understanding of how genotype mediates the interactions between phytoplasma infection and insect herbivores, future studies should assess additional wild and cultivated cranberry genotypes.

### Do phytoplasma infection and genotype affect a belowground herbivore?

As pointed out by Grunseich *et al.* (2020), we are not aware of any investigations addressing how alterations in root chemistry by phytoplasma infection may impact the performance and behavior of root‐feeding herbivores. Ours is the first case study demonstrating that phytoplasma infection can benefit a subterranean herbivore, such as *A. orientalis*. Larvae of *A. orientalis* exhibited higher survival when fed phytoplasma‐infected cranberry roots compared to those fed uninfected roots (Fig. 2A, B). Interestingly, although larval consumption was reduced (Fig. 2E), their size increased when feeding on infected roots (Fig. 2C, D), ultimately leading to a higher ECI (Fig. 2F). These findings suggest that the roots of infected plants offer improved nutritional quality to *A. orientalis*. While genotype had minimal impact on *A. orientalis* larval survival and growth, it did influence their consumption. Moreover, there was a negligible interaction between genotype and phytoplasma infection on *A. orientalis*. Notably, “Crimson Queen” appeared to have a more pronounced effect on *A. orientalis* larval weights and ECI compared to “Ben Lear,” despite the phytoplasma titer being higher in “Ben Lear” roots than in “Crimson Queen” roots.

### Do phytoplasma infection and genotype affect plant traits?

As expected, the growth (length and weight) of shoots (Fig. 3A, B) and roots (Fig. 4A) of cranberries infected by phytoplasma was reduced compared to uninfected plants. Phytoplasmas are known to regulate various hormones associated with growth and development in plants, including ethylene, auxin, cytokinins, gibberellin and abscisic acid (Chang, 1998; Dermastia, 2019). In cranberry, phytoplasma infection influenced the regulation of genes associated with developmental pathways (Pradit *et al.*, 2019b), which might be involved in shoot and root growth. Interestingly, the weights of shoots (Fig. 3B) and roots (Fig. 4A) of the genotype “Crimson Queen” were not affected by infection, suggesting that plants bred for higher productivity might partially compensate in size. Regardless, phytoplasma‐infected plants will not produce fruit; thus, the implications of this compensation remain unclear.

Recent studies have documented changes in the chemical composition of both leaves and roots in plants as a consequence of phytoplasma infection (e.g., Al‐Yahyai *et al.*, 2014; Kiprovski *et al.*, 2018; Hemmati & Nikooei, 2019; Raiesi & Golmohammadi, 2020; Görg *et al.*, 2021). For example, infection of Mexican lime (*Citrus aurantifolia* Swingle) by the phytoplasma “*Candidatus* Phytoplasma aurantifolia” (16SrII‐B subgroup) resulted in higher soluble carbohydrates and starch in the leaves but lower levels in the roots. Additionally, phenolic levels increased while nitrogen levels decreased in infected leaves (Raiesi & Golmohammadi, 2020). In another study, Kiprovski *et al.* (2018) showed that infection of evening primrose (*Oenothera biennis* L.) by the phytoplasma “*Candidatus* Phytoplasma solani” (16SrXII‐A subgroup) led to increased peroxidation of lipids, phenylalanine ammonia‐lyase activity, total sugar, polyphenols, and anthocyanins content in the leaves. However, there was a decrease in photosynthetic pigments and total flavonoids, while only the total polyphenol content increased in the roots. These reported changes in shoot and root chemistry can be attributed to altered source‐sink relationships caused by phytoplasma infection (Lepka *et al.*, 1999), leading to movement of nutrients such as sugars and amino acids from source tissues to sink tissues (Bertaccini, 2007). In a prior study, we showed elevated nitrogen levels and reduced proanthocyanidin levels in cranberry (“Crimson Queen”) infected by phytoplasma (Pradit *et al.*, 2019a). We extended these previous studies by demonstrating that these effects are consistent across two cranberry genotypes (“Ben Lear” and “Crimson Queen”) and that they occur in both leaves (Fig. 3C–E) and roots (Fig. 4B–D) of plants. Phytoplasma infection is known to alter the regulation of phytohormones associated with antimicrobial and antiherbivore defenses, such as salicylic acid and jasmonic acid (Dermastia, 2019). Generally, these studies show that infection causes an upregulation of genes associated with the salicylic acid pathway and a downregulation of those associated with the jasmonic acid pathway (Dermastia, 2019). This pattern was also observed in cranberry (Rodriguez‐Saona *et al.*, 2021) and could explain the reduced levels of proanthocyanidins in infected plants, as these are regulated by the jasmonic acid pathway (An *et al.*, 2015; An *et al.*, 2021).

### Implications and future directions

From an ecological perspective, infection by phytoplasma in cranberries could lead to increased competition for high‐quality resources, i.e., plants with increased nutrients and lower defenses, among both above‐ and belowground insect herbivores. This competition may be indirectly mediated by the induced responses of these herbivores. For instance, nonvector herbivores such as *L. dispar* and *A. orientalis* may compete with the vector *L. vaccinii* either directly by removing resources or indirectly by inducing defenses in plants, thereby potentially altering the plant–herbivore–pathogen interactions. From an applied perspective, high levels of phytoplasma infection could exacerbate pest problems in cranberry beds by increasing the susceptibility of plants to a community of insect herbivores both above‐ and belowground. This may necessitate increased levels of pest monitoring and more rigorous control measures.

Certain gaps in our knowledge still remain, requiring further exploration. In this study, we used infected plants from the field due to a lack of knowledge regarding the time required for the vector to acquire and transmit the disease. This approach may have resulted in variable levels of infection in tissues. Consequently, the potential impact of these varying infection levels on herbivores requires further investigation. Moreover, in our study, we analyzed the changes in phytochemistry resulting from phytoplasma infection in the absence of herbivory. Therefore, the investigation of whether phytoplasma infection mediates interactions between above‐ and belowground herbivores through induced responses in plants will be the focus of future research. Finally, to understand how this knowledge can aid in controlling the spread of false blossom disease in cranberry, it is crucial that future studies develop methods that prevent the phytoplasma from manipulating the host‐plant or that enhance host‐plant resistance to the disease and herbivores.

## Conclusion

**Fig. 6:**
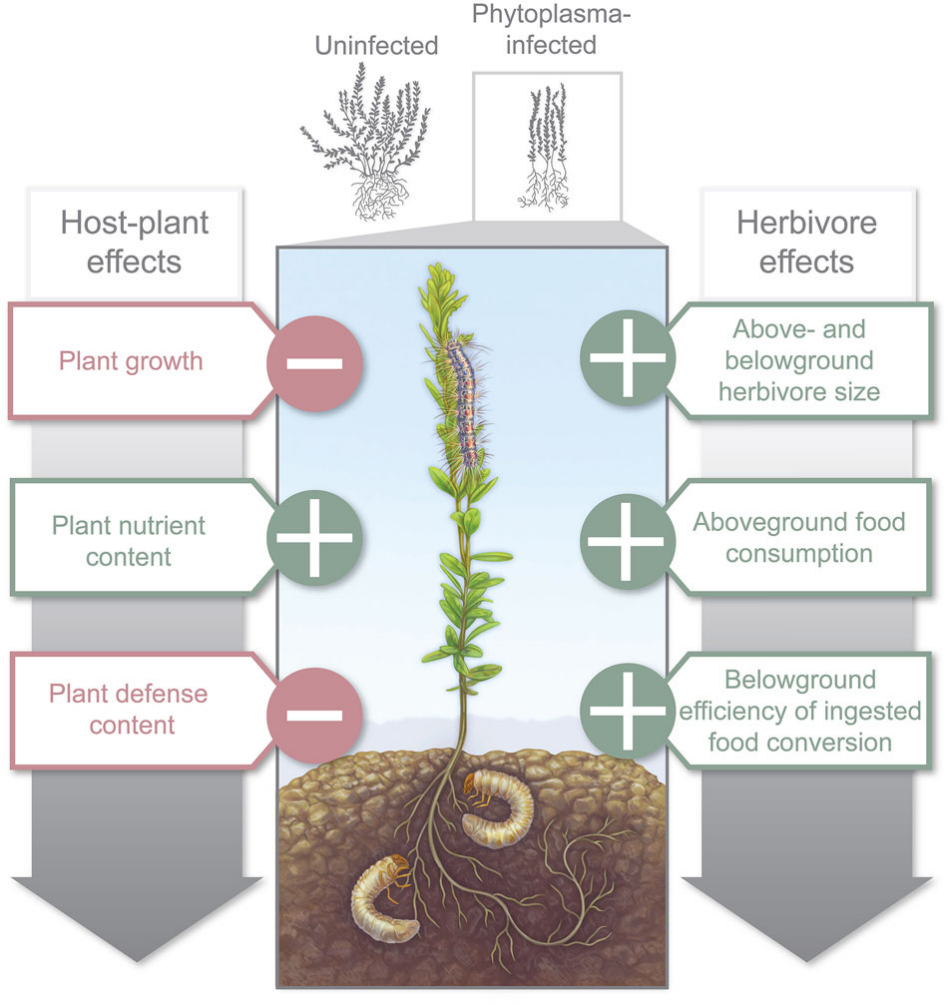
Schematic illustration summarizing the effects of phytoplasma infection on the performance and feeding behavior of above‐ and belowground insect herbivores as well as its effects on shoot and root traits in cranberries. The “+” symbol indicates a positive effect, while the “−” symbol indicates a negative effect.

Consistent with the “host manipulation” hypothesis, this study demonstrates that phytoplasma infection induces physical and chemical changes in cranberry, manifesting as stunted growth, increased nutrient status, and reduced defenses (Fig. 6). These alterations, seemingly for the pathogen's benefit, were also found to enhance the performance and feeding behavior of both above‐ and belowground insect herbivores (Fig. 6). Furthermore, we established that, although the strength of these effects varied in some instances, they remained largely consistent across two host‐plant genotypes. This study offers new insights into how different genotypes influence phytoplasma manipulations and their effects on nonvector organisms, both above‐ and belowground.

## Disclosure

We declare that the authors have no conflict of interest.

## Supporting information


**Table S1** Primers and probes used for qPCR assay.
