# Supplementary material for: Phytoplasma infection renders cranberries more susceptible to above‐ and belowground insect herbivores
Source: Insect Sci. 2024 Sep 15;32(3):957–72. doi: 10.1111/1744-7917.13444 (PMC12175984; doi:10.1111/1744-7917.13444)
Supplement: Supplementary file 1 — Table S1 Primers and probes used for qPCR assay. [file INS-32-957-s001.docx]

**Table S1.** Primers and probes used for qPCR assay.

| Target gene | Prime/Probe | Sequence |
| --- | --- | --- |
| *secY* | F | GCG GTA GGA TTG GCT TTA TCT |
|  | R | GCT AAT GAA AGC TCC TAC CAC T |
|  | Probe | /56-FAM/TCA ACG TGA TGG AAT CTC CTC GCT /3BHQ_1/ |
| *Actin* | F | CGC CCT CAT GAA GAT CCT TAC |
|  | R | CTT TGC AGT CTC CAG CTC TT |
|  | Probe | /56-FAM/AGC TTG CAT ACG TGG CTC TTG ACT /3BHQ_1/ |
| *RNA Helicase 8* | F | ACG TCT GGT GTA GCA CAT TC |
|  | R | GGC ATG AGA AGA ATG AGA GAG G |
|  | Probe | /56-FAM/TAG GAC CCA CAA TTA GCA CAA GGC C/3BHQ_1/ |
